# Low Levels of Dehydroepiandrosterone Sulfate in Younger Burnout Patients

**DOI:** 10.1371/journal.pone.0140054

**Published:** 2015-10-06

**Authors:** Anna-Karin Lennartsson, Töres Theorell, Mark M. Kushnir, Ingibjörg H. Jonsdottir

**Affiliations:** 1 Institute of Stress Medicine, Gothenburg, Sweden; 2 Center for Psychiatry Research, Department of Clinical Neuroscience, Karolinska Institute, Stockholm, Sweden; 3 Stress Research Institute, Stockholm University, Stockholm, Sweden; 4 ARUP Institute for Clinical and Experimental Laboratory, Salt Lake City, Utah, United States of America; Leiden University Medical Centre, NETHERLANDS

## Abstract

**Objective:**

Dehydroepiandrosterone sulphate (DHEA-s) is an anabolic protective hormone of importance for maintenance of health. DHEA-s levels peak in young adults and decline thereafter with age. DHEA-s has previously been shown to be lower in individuals reporting prolonged stress. This study investigates DHEA-s levels in patients with clinical burnout, a disorder caused by long-term psychosocial stress.

**Methods:**

122 patients (51% men) and 47 controls (51% men) in the age 25–54 years were included in the study. DHEA-s levels were compared between patients and controls in the whole sample and within each of the three 10-year-interval age groups.

**Results:**

In the youngest age group (25–34 years), DHEA-s levels were on average 25% lower in the patients (p = 0.006). The differences in DHEA-s levels between patients and controls were more pronounced among female than male participants (on average 32% and 13% lower, respectively). There were no differences in DHEA-s levels between patients and controls in the age group 35–44 years (p = 0.927) or 45–54 years (p = 0.897) or when analyzing all age groups together (p = 0.187).

**Conclusion:**

The study indicates that levels of the health promoting “youth” hormone DHEA-s are low in younger burnout patients. The fact that younger adults have much higher DHEA-s levels and more pronounced inter-subject variability in DHEA-s levels than older individuals might explain why burnout status differentiates patients from controls only among the youngest patients included in this study.

## Introduction

Burnout can be defined as a negative affective state consisting of emotional exhaustion, cognitive weariness and physical fatigue, which is caused by chronic psychosocial stress [1]. It leads to reduced work ability [2] and often also long-term sick leave [3]. Burnout is often accompanied with symptoms of depression and anxiety. Besides the fatigue and mental health burden, burnout is associated with increased risk of adverse physical health [4, 5]. The present study investigates the levels of dehydroepiandrosterone sulfate (DHEA-s) in patients with clinical burnout.

In addition to being a precursor to androgens and estrogens, DHEA-s is an active hormone with effects on its own [6]. DHEA-s has a regenerative and protective role important for maintenance and restoration of health. For example, DHEA-s enhances the activity of the body’s own antioxidant system, prevents atherosclerosis by stimulating the production of NO in the vessel and stimulates neurite growth, neurogenesis, and neural survival [6]. DHEA-s is produced in the zona reticularis area of the adrenal cortex in response to adrenocorticotrophic hormone (ACTH). The production peaks in young adulthood, thereafter the levels decline progressively [7] by 2–4% per year.

Low levels of DHEA-s have mostly been reported to be associated with, independently of age, both subjective perception of poor health [8] and different disease states [9–12]. We and others have previously reported that DHEA-s levels are lower in individuals reporting psychosocial stress [13, 14]. Since burnout is a consequence of long-term stress, and since it is a syndrome associated with both psychological and physiological adverse health, it would be of interest to investigate DHEA-s levels in burnout. Low DHEA-s levels in these patients could constitute one of the links between stress and adverse health associated with burnout. However, out of four previous publications on DHEA-s levels in burnout, three reported no difference between burnout subjects and controls [15–17] and one publication reported higher DHEA-s levels in the individuals with burnout [18]. The present study aims to investigate DHEA-s levels in patients with clinical burnout compared to healthy controls without burnout symptoms. The relatively large sample of available patients in the study allows for separate analyses in different age groups, which is an advantage since DHEA-s is strongly age related.

## Method

### Participants

122 patients with clinical burnout (62 men; 60 women) and 47 healthy controls (24 men and 23 women with clear absence of burnout symptoms) in the age 25–54 years were included in the study.

The patients were recruited from a specialist clinic, which exclusively treats patients with stress-related mental disorders, in the region of Västra Götaland, Sweden. All the patients fulfilled the diagnostic criteria for stress-related exhaustion disorder as previously described [14]. Stress-related exhaustion disorder is a criteria-based diagnosis that has been used in Sweden since 2005 to define patients seeking health-care for clinical burnout. With the aim to study DHEA-s levels in these patients, selection of patients was performed among those who were between 25 and 54 years, had answered the questionnaire regarding burnout symptoms, depression and anxiety at all time-points up to one year after inclusion, thus at the initial visit and at the 3, 6 and 12 months follow up, and had available serum samples in the freezer. A total of 63 male patients fulfilled the criteria described above. Among the 141 females who fulfilled the criteria, 63 female patients were randomly selected in order to include an equal number of men and women. One of the male patients and three of the female patients had samples that were not successfully analyzed. In the present study, we report the DHEA-s levels at baseline for these 122 patients. 30% of the patients were taking antidepressants.

Blood samples and burnout scores from 198 individuals in the age 25–54 years were available from another study to be included as control subjects, which explains the age criteria for the patient group. These individuals were recruited with the aim of studying the relationship between biological markers of stress and perceived stress. Before inclusion, the subjects underwent a screening test, including anthropometric measurements and obtaining blood samples to ensure the following exclusion criteria; having a body mass index less than 18.5 kg/m2 or over 30 kg/m2, high blood pressure, infection, vitamin B–12 deficiency (high homocysteine), known systemic disease such as diabetes or thyroid disease, known psychiatric disease or alcohol abuse. Women taking estrogens as well as nursing, pregnant and postmenopausal women were not included. Subjects who were taking psychoactive medications or any medications that may affect the hypothalamus–pituitary–adrenal (HPA) axis function were not included. Among the 198 individuals available for this study to serve as control group, only individuals without symptoms of burnout identified using the Shirom-Melamed Burnout Questionnaire [1] (scores lower than 2.0) were included in the present study as controls (24 men; 23 women).

Fifteen percent of the controls and 20% of the patients were nicotine users. One of the controls and 11% of the patients had a risky high alcohol consumption according to the Alcohol Use Disorders Identification Test (AUDIT) (scores 8–15) [19]. None had scores indicating alcohol abuse (>15) since this was one of the exclusion criteria in the study from which the control subjects were drawn. Alcohol abuse according to AUDIT was also a differential diagnostic criterion for exhaustion and these patients were not accepted at the stress clinic and thus not included in this study. Both the patient group and the control group consisted of 51% men. Aiming on separate comparisons of DHEA-s between patients and controls in different ages (see statistical analyses section below), three 10-year-interval age groups were created. Age group 25–34 years included 29 patients (41% men) and 14 controls (57% men), age group 36–45 years, 49 patients (55% men) and 11 controls (46% men) and finally age group 46–54, 44 patients (52% men) and 22 controls (50% men). Time periods for recruitment were between 2004 and 2010 for the patients and between 2004 and 2008 for the controls. All participants gave written informed consent. The study was approved by the Regional Ethical Review Board in Göteborg, Sweden, and was conducted according to the Helsinki Declaration.

### Hormone assay

Blood samples (in total 65.6 ml) were drawn in the morning between 7.30 and 10 from an antecubital vein. The participants had fasted overnight and were instructed to abstain from hard physical exercise for 24 hours prior to the blood sampling. The samples were collected in two different tubes; pre-chilled tubes containing EDTA and serum separator tubes. The serum separator tubes were centrifuged during 10 minutes in 20°C with a speed of 3500 rpm (with a Sigma 2–16 KC centrifuge) and the samples were thereafter stored at 80°C until assayed. Serum concentration of DHEA-s was measured by quantitative electro-chemiluminescent immunoassay (Roche Diagnostics Corporation). Inter-assay coefficient of variation was below 12%.

### Statistical analyses

Pearson correlation analyses were performed between DHEA-s levels and age in the whole sample and in patients and controls separately. T-test was used to compare DHEA-s levels between men and women in the whole sample as well as in patients and controls separately. Partial correlation analyses were performed between DHEA-s levels and BMI separately in men and women, while adjusting for age. To compare DHEA-s levels between patients and controls, t-tests were performed first in the whole sample and then within each of the three age groups. In addition, the t-tests were redone without the patients that were taking antidepressants. Since drinking alcohol and smoking may affect DHEA-s levels [20, 21] the t-tests comparing DHEA-s levels between patients and controls were also performed without these individuals.

## Results

### DHEA-s in relation to age, sex and BMI

As expected, DHEA-s levels were negatively associated with age (total: r = -0.34, p < 0.001), although the correlation was stronger in controls than in patients (controls: r = -0.50, p <0.001; patients: r = -0.26, p = 0.004). Also as expected, DHEA-s levels were higher in males than in females (total group: 6.72 and 4.63, respectively, p < 0.001; patients: 6.52 μmol/L and 4.52 μmol/L, respectively, p < 0.001; controls: 7.23 μmol/L and 4.93 μmol/L, respectively, p = 0.004). DHEA-s levels were not associated with BMI after adjusting for age (men: p = 0.493; women: p = 0.409).

### Comparisons of DHEA-s levels in patients and controls

**Fig 1:**
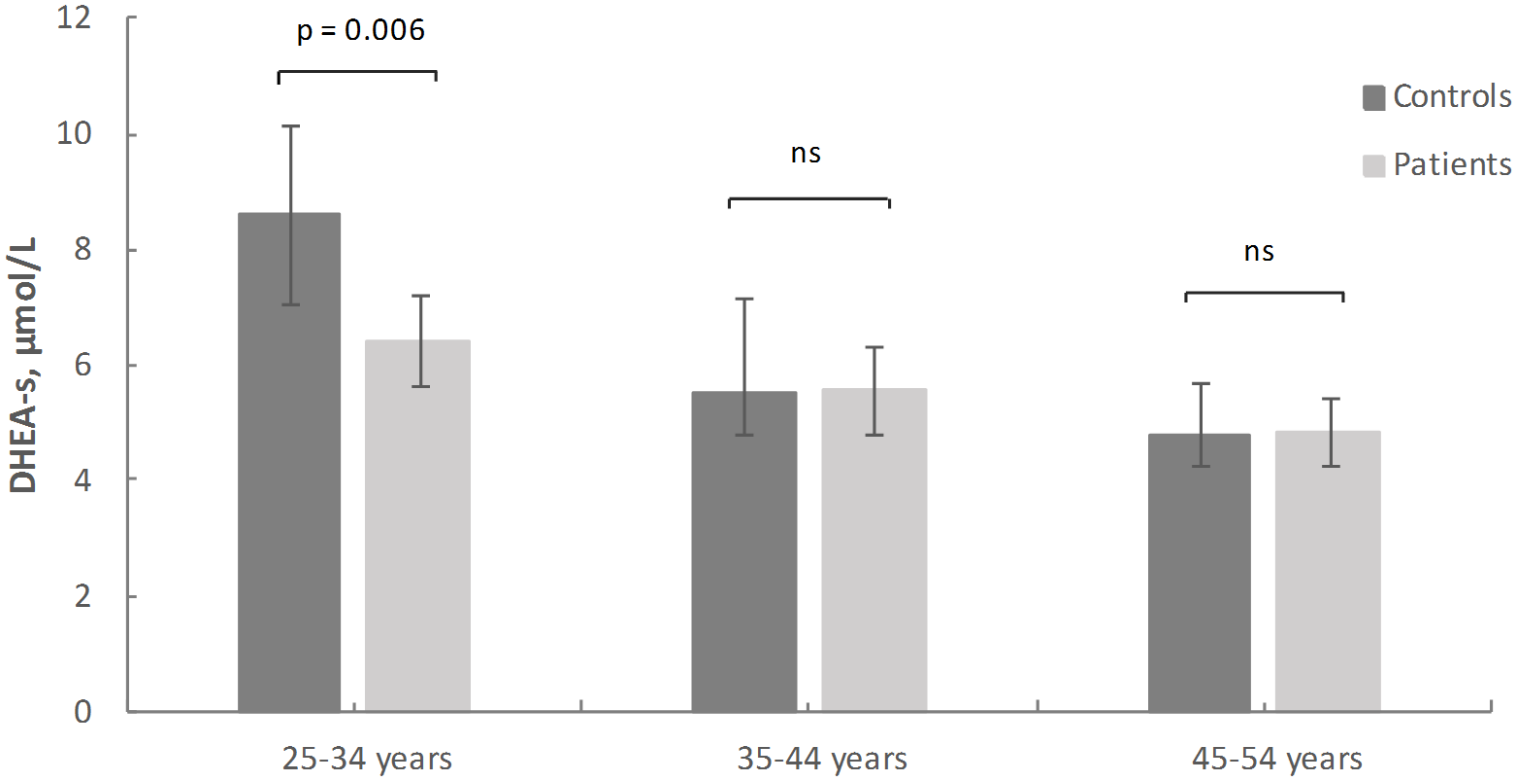
Mean (95% confidence intervals) DHEA-s levels in patients and controls in the three age groups.

In the youngest age group (25–34 years), DHEA-s levels were on average 25% lower in the group of the burnout patients than the controls (p = 0.006). The difference in DHEA-s levels between patients and controls were more pronounced among female than among male subjects (on average 32% and 13% lower, respectively). There were no differences in DHEA-s levels between patients and controls in the age groups 35–44 years (p = 0.927) and 45–54 years (p = 0.897). The DHEA-s levels in burnout patients and controls in the different age groups are displayed in Fig 1. When the three age groups were analysed together, there was no significant difference in DHEA-s levels between the patients and controls (5.54 vs 6.10 μmol/L, p = 0.187). Excluding the patients that were taking antidepressants did not change the results (data not shown).

Since alcohol consumption and smoking may affect DHEA-s levels additional comparisons without these individuals were performed. Restricting the analyses to non-nicotine users (patients = 92, 51% men, mean age = 40.7 years; controls = 39, 51% men, mean age 40.3 years) did not change the results (data not shown). Restricting the analyses to participants without risky alcohol consumption (< 8 on AUDIT) (patients = 103, 48% men, mean age = 40.9 years; controls = 46, 50% men, mean age 40.5 years) showed a trend to lower DHEA-s levels in the patient group than in controls (p = 0.058), but otherwise the same results, thus, lower DHEA-s in patients only in the youngest age-group when analyzing age groups separately (data not shown).

## Discussion

The results showed that, in the youngest group (25–35 years), DHEA-s levels were significantly lower in the patients than in the healthy controls, but among the other age groups (36–45 and 46–54 years) there were no differences in DHEA-s levels between patients and controls. When restricting the analysis to only participants without high-risk alcohol consumption, a trend to difference between patients and controls in the whole group was revealed, thus lower DHEA-s in the patients. However, this difference was still due to the large difference between patients and controls among the younger participants. It is likely that low DHEA-s levels are primarily observed in younger adults with clinical burnout since the DHEA-s levels are much higher and the inter-subject variability is larger in younger individuals. Thus, a plausible interpretation is that depressed DHEA-s levels in patients with clinical burnout are more likely among younger individuals although changes in these hormonal levels could be present even among older patients.

Four previous publications report DHEA-s level comparisons between individuals with and without burnout. These studies include both clinically diagnosed patients [16] and “high burnouts” defined using burnout scales [15, 17, 18]. Three of these studies found no difference in DHEA-s levels between burnout-subjects and controls [15–17] while one study found higher DHEA-s levels in the burnout group [18]. One plausible explanation why no differences were seen between the groups in some of the previous studies could be that there were large variations in age among the study subjects. For instance, in one of the studies age ranged from 27 to 65 years [18] and in another from 19 to 59 years [16]. In the present study, the number of burnout subjects included was larger than in the previous studies. The relatively large number of patients allows for separate analyses in different age groups. Our age specific analysis revealed a difference only in the youngest age group. Low DHEA-s levels in these patients are likely an effect of long-term stress exposure. During prolonged psychosocial stress, steroid biosynthesis may be shifted from synthesis of adrenal androgens to corticosteroid pathways to ensure maintenance of cortisol production [22, 23].

There are several factors that may affect DHEA-s levels and all cannot be controlled for. This limits the interpretation of the results in this study. The well-known and important factors age and sex are controlled in this study since the participants are divided into age groups and since there is equal distribution of men and women in the groups. Factors that may affect DHEA-s levels such as alcohol consumption [21], nicotine use [20], body mass index and use of medication such as antidepressants have been taken into consideration in this study. Furthermore, many factors that could affect DHEA-s levels are also considered since they were exclusion criteria for both patients and controls. This includes diseases such as thyroid disease, obesity and alcohol abuse.

In conclusion, the present study indicates that levels of the health promoting “youth” hormone DHEA-s are low in younger burnout patients. The fact that younger adults have much higher DHEA-s levels and more pronounced inter-subject variability in DHEA-s levels than older individuals might explain why burnout status differentiates patients from controls only among the youngest. The results should be confirmed by other studies. Low DHEA-s levels in these patients could constitute one of the links between stress and adverse health associated with burnout.

## Supporting Information

S1 Dataset DHEA-s levels in clinical burnout. (SAV)
