# Correction: Low Levels of Dehydroepiandrosterone Sulfate in Younger Burnout Patients

**DOI:** 10.1371/journal.pone.0143192

**Published:** 2015-11-12

**Authors:** Anna-Karin Lennartsson, Töres Theorell, Mark M. Kushnir, Ingibjörg H. Jonsdottir

There is an error in affiliation 4 for author Mark M. Kushnir. Affiliation 4 should be: ARUP Institute for Clinical and Experimental Pathology, Salt Lake City, Utah, United States of America.
